# Point of care ultrasound measurement of paralumbar caudal vena cava diameter and caudal vena cava to aortic ratio in hypovolemic dogs

**DOI:** 10.3389/fvets.2024.1467043

**Published:** 2024-10-28

**Authors:** Jenna H. Cardillo, Kristin M. Zersen, Amanda A. Cavanagh

**Affiliations:** Department of Clinical Sciences, College of Veterinary Medicine and Biomedical Sciences, Colorado State University, Fort Collins, CO, United States

**Keywords:** volume assessment, point of care ultrasound, caudal vena cava, fluid therapy, dog

## Abstract

**Background:**

Accurate assessment of intravascular volume is critical for precise fluid prescription. In people, bedside or point of care ultrasound is used to measure the inferior vena cava, with or without paired aortic measurement, to estimate intravascular volume.

**Objective:**

To determine if point of care ultrasound measurement of the caudal vena cava (CVC) diameter or the CVC diameter to the abdominal aorta (Ao) diameter (CVC:Ao) at the paralumbar view are associated with changes in intravascular volume, mean arterial pressure (MAP), or cardiac output in normovolemic and hypovolemic dogs.

**Animals:**

8 purpose-bred dogs.

**Methods:**

Pressure-targeted hemorrhagic shock was induced in purpose-bred dogs under general anesthesia. Dogs were exsanguinated to a mean arterial pressure of 40 mmHg, or a maximum 60% blood volume lost, then auto-transfused shed blood. At a left paralumbar view, longitudinal plane measurements of the abdominal CVC diameter and aortic diameter were obtained. Measurements were performed at 4 timepoints: baseline under anesthesia (TP1), after hemorrhagic shock was induced (TP2), after ½ of shed blood had been re-transfused (TP3), and post-resuscitation with completed re-transfusion (TP4). Additional variables collected included cardiac output using thermodilution and arterial blood pressure.

**Results:**

CVC:Ao was not significantly different between timepoints and was not associated with changes in CO (*p* = 0.28) or MAP (*p* = 0.50). CVC diameter was significantly different between baseline (TP1) and hemorrhagic shock (TP2). CVC diameter was significantly different at TP2 compared to TP1 after controlling for the effect of CO (*p* = 0.03) and MAP (*p* = 0.001). Aortic diameter was also significantly different at TP2 (*p* = 0.002, p = 0.001) and TP3 (*p* = 0.023, *p* = 0.017) compared to TP1 after controlling for CO and MAP.

**Conclusions and clinical importance:**

Obtaining point of care ultrasound images for CVC:Ao measurement was feasible. With a marked decrease in intravascular volume, both CVC and Ao diameter decreased, resulting in an unchanged CVC:Ao. Despite changes in CVC and Ao diameters, these changes were not associated with measured changes in CO, emphasizing that CO is not a direct estimate of intravascular volume and is affected by many compensatory mechanisms. Additional studies are needed to determine the most accurate method for bedside measurement of intravascular volume status.

## Introduction

Accurate assessment of intravascular volume and fluid responsiveness is vital in critically ill patients. While often used interchangeably, these parameters are distinct though interrelated (1). Intravascular volume status can be assessed using static parameters including physical examination findings, measured blood pressure, lactate, central venous pressure, and pulmonary arterial occlusion pressure (PAOP) (2). There are limitations to the use of static parameters including the need for invasive catheterization for PAOP and lack of accuracy due to compensatory mechanisms associated with hypovolemia (3). Dogs’ robust ability to compensate for hypovolemia and maintain normotension represents a key confounding factor in intravascular volume assessment. As a result, single cardiac output and MAP measurements are unlikely to have a linear relationship with intravascular volume status (3).

Dynamic parameters are useful in predicting fluid responsiveness by revealing the change in stroke volume in response to a maneuver that increases venous return and preload, such as endogenous or exogenous fluid boluses or preload altering changes in intrathoracic pressure (1, 3, 4). Fluid responders are patients that have a 10–15% increase in cardiac output in response to an increase in preload. Dynamic parameters include stroke volume variation, pulse pressure variation, systolic pressure variation, plethysmographic variability index, and cardiac output measurement (1, 3). These dynamic variables are most accurate in mechanically ventilated patients because the depth of the breath and effect on preload is controlled with each respiratory cycle (3). The need for mechanical ventilation and the need for central catheter placement limits the usefulness of these parameters in emergency veterinary patients.

Point-of-care ultrasound can provide static and dynamic parameter measurements in a bedside, non-invasive, repeatable manner. The accuracy of these measurements is not well established in dogs (1). Static POCUS parameters for assessing intravascular volume include measurement of left ventricular end-diastolic diameter, caudal vena cava diameter, and caudal vena cava to aorta ratio (CVC:Ao) (1). With intravascular volume loss, the caudal vena cava diameter decreases as this low-pressure vessel collapses with decreased transmural pressure. While CVC diameter must be referenced to patient body size, using aortic diameter to create a ratio allows for assessment without body weight specific reference ranges. The use of CVC:Ao for volume assessment is based on a changing CVC diameter with changes in intravascular volume, while aortic diameter remains relatively unchanged. The caudal vena cava collapsibility index (CVCCI) is a dynamic POCUS parameter used to assess volume responsiveness. The CVCCI is measured as the abdominal caudal vena cava crosses the diaphragm and is defined as (CVC_max_ –CVC_min_)/CVC_max_, representing the dynamic change of the CVC with the respiratory cycle (5). Previous studies have shown poor inter-rater variability and therefore reference values for this measurement have not been successfully established in normal dogs (5). Studies attempting to predict fluid responsiveness using CVCCI have discrepant results (6, 7).

Intravascular volume assessment and fluid responsiveness using point of care ultrasound techniques is effective and accurate in people (8–13). Measurement of the inferior vena cava (IVC) diameter reflects intravascular volume and its collapsibility through the respiratory cycle in ventilated patients (IVCCI) accurately predicts fluid responsiveness in people (11, 14, 15). In dogs, studies have investigated static and dynamic POCUS measurements of the CVC at various locations, in various planes, in both experimental and clinical patients with various volume derangements (16–19). Specifically, measurements of the CVC, aorta, and CVC:Ao have been performed at intercostal (5, 7, 16) and paralumbar locations (5, 17–19), in normal dogs (7), with naturally occurring shock (17), and in the settings of blood loss (18–20) and diuretic induced volume loss (16). These varied study designs have yielded varying results. In the setting of blood loss, two studies found a significantly decreased CVC:Ao (19, 20), while another did not (18).

More data is needed in dogs regarding the significance of CVC, Ao, and CVC:Ao measurements in volume assessment and their relationship with both static and dynamic monitoring parameters. No existing studies have compared CVC and Ao measurements with specific, controlled changes in volume status, in conjunction with cardiac output monitoring by the gold standard thermodilution. Investigation of the interaction between hemodynamic parameters and ultrasound measurements in various shock states may shed further light on cardiovascular physiologic responses. Further, because there are various techniques and approaches, more data is needed to determine which location and plane is most clinically useful and accurate between operators.

The purpose of this study was to determine if static parameters, CVC diameter and CVC:Ao at the paralumbar view are associated with changes in intravascular volume, MAP, or cardiac output. We hypothesized that POCUS measured CVC diameter and CVC:Ao would be associated with intravascular volume. However, due to compensation and the indirect relationship between volume status, CO, and MAP, we hypothesized that POCUS measurements would not be associated with MAP or cardiac output in experimentally induced hypovolemia.

## Materials and methods

Eight purpose-bred female spayed beagles were used in this study. Each dog was determined to be healthy based on physical examination, complete blood count, and serum biochemistry panel. All procedures were approved by the Institutional Animal Care and Use Committee at Colorado State University (IACUC #2237).

Dogs were pre-medicated with hydromorphone (0.1 mg/kg IM) and an IV catheter was placed in a cephalic vein. Dogs were preoxygenated with 100% fraction of inspired oxygen (FiO_2_) administered by facemask. General anesthesia was induced with propofol (5–10 mg/kg IV) to effect. Dogs were orotracheally intubated, and anesthesia was maintained using isoflurane vaporized in 100% FiO_2_ and delivered via a circle system. All dogs were spontaneously breathing throughout the study.

A jugular catheter was placed in a jugular vein and an arterial catheter was placed in a dorsal metatarsal artery. A Swan-Ganz catheter was placed using a flow-directed technique and location in the pulmonary artery was confirmed via pressure waveform analysis. All catheters were aseptically placed. Standard, continuous monitoring was employed throughout the procedure, including ECG, pulse oximetry, esophageal temperature, and end-tidal partial pressure of carbon dioxide via sidestream sampling. A forced air warming device and heated water blanket were used to maintain normothermia.

After induction of anesthesia and instrumentation, the mean arterial pressure (MAP) was stabilized at 70–80 mmHg for 10 min before images were obtained (TP1). Blood was then manually removed from the jugular catheter over 20 min until a MAP of 40 mmHg was reached or a maximum of 60% blood volume (blood volume = 90 mL/kg) was removed, whichever point was reached first. Shed blood was collected in blood collection bags containing citrate phosphate dextrose adenine. After 10 min, images were obtained (TP2). Then 50% of the volume of shed blood was re-transfused over 15 min. Ten minutes later, images were obtained (TP3). The remaining 50% of blood was re-transfused over 15 min, and after 10 min, final images were obtained (TP4). Dogs that had a 10% or greater increase in CO after re-transfusion were classified as fluid responders. Using point of care ultrasound, the CVC diameter and aortic diameter in a longitudinal plane at the left paralumbar view were measured at each timepoint. Because the respiratory cycle minimally affects the diameter of the CVC and Ao at this location, single CVC and Ao measurements were utilized as described previously (5). Cardiac output measurement was performed at each timepoint using thermodilution according to manufacturer guidelines.^1^ All CO measurements were obtained in duplicate with 2–5 min between measurements.

### Ultrasound examination and measurement

Probe operation and B mode image acquisition were performed by the same investigator (JC) for each dog. Dogs were positioned in right lateral recumbency, and the abdominal CVC and aortic diameter were imaged using an ultrasound system^2^ equipped with a 5–10 mHz microconvex probe. Care was taken to apply minimal pressure to avoid change in vessel diameter due to excessive external probe pressure. Once the vessels were visualized another investigator (AC) captured and labeled B mode 7 s cine loops for post-hoc measurements. Vessel diameter measurements perpendicular to the vessel walls were obtained in B mode using electronic calipers incorporated in the DICOM viewer^3^ using inner edge to outer edge technique. CVC measurements were performed after frame-by-frame analysis to determine the maximal CVC diameter. Vessel diameter measurements were performed by a single investigator (JC). While the investigator was not blinded during the study (while collecting cine loops), they did not have access to the CV parameters during the viewing of the cine loops or measurements. Both operators involved in this study had a minimum of 4 years of experience with POCUS.

**Figure 1:**
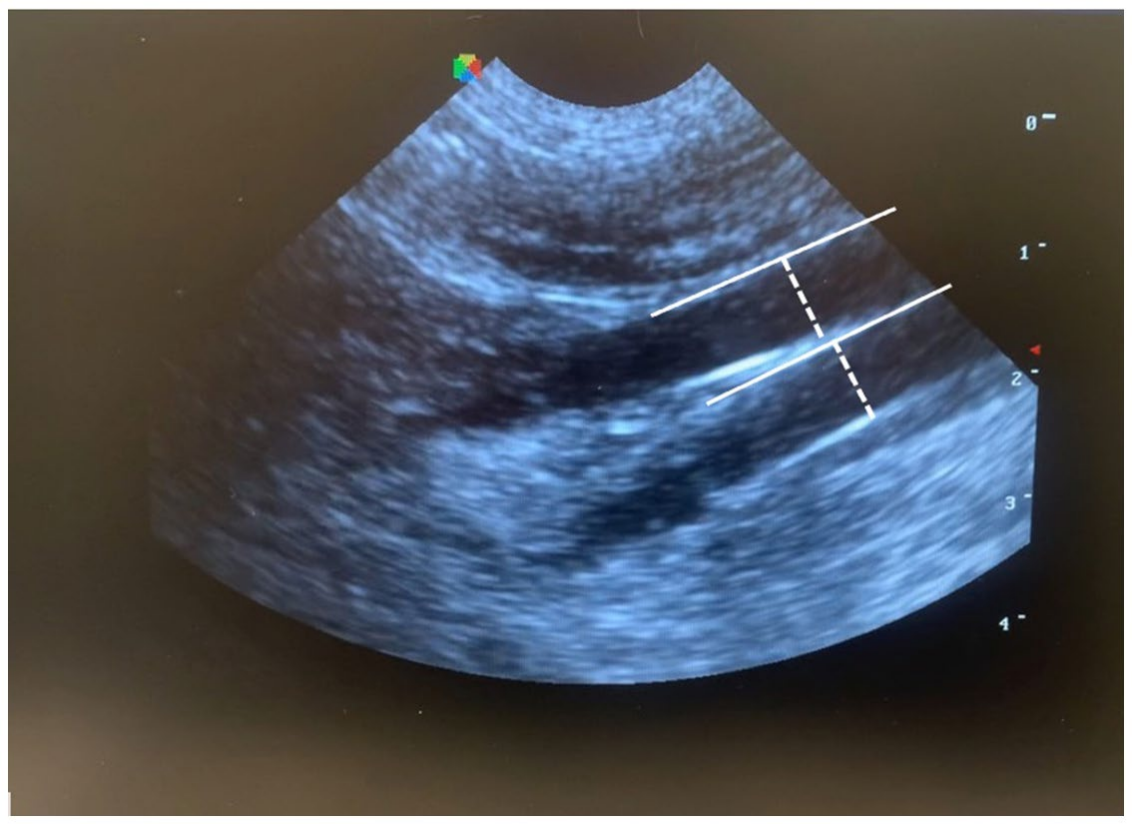
Measurement of CVC:Ao at a left paralumbar view in B-mode, by measuring the diameter perpendicular to the wall of each vessel (CVC:Ao, caudal vena cava to abdominal aorta ratio).

For CVC diameter and CVC:Ao measurements, the transducer was placed just caudal to the left 13th rib then angled cranially, to visualize the left kidney. The probe was then slowly fanned medially and caudally until the CVC and Ao were visualized in parallel in the same longitudinal plane; diameter was measured for both vessels perpendicular to the vessel wall in the same plane (Figure 1).

## Statistics

Continuous data was described using means and standard deviation. Outcome data was evaluated for normality using Shapiro–Wilk statistics. If normality was not met, data was transformed into log scale. Analysis was performed using a linear mixed model with the factor and timepoint and an interaction effect. If the interaction term was not significant or the main term was not significant, the interaction term was excluded and data was analyzed with the factor and timepoint to calculate adjusted effects. A *p*-value of 0.05 was used to determine statistical significance. SAS^4^ was used for all statistical analyses.

## Results

This study was conducted on 8 female spayed purpose-bred beagles. Descriptive statistics were performed for all 8 dogs (*n* = 8). Dog 1 was excluded from image related descriptive statistics and linear mixed model analysis due to loss of image data. Normal data is presented as mean ± standard deviation. If normality was not met, data is reported as median (range). Median age was 5.0 years (3.0, 5.0). The mean weight was 8.83 ± 1.56 kg. The mean volume of blood withdrawn to induce hypovolemic shock was 40 ± 12 mL/kg.

Cardiac output was significantly lower at TP2 (hemorrhagic shock) compared to TP1 (baseline) (*p* < 0.001). Cardiac output was not different between TP1 and TP4 (re-transfusion of shed blood) (p 0.71). MAP was significantly lower at TP2 (hemorrhagic shock) compared to TP 1 (baseline; p 0.018) (see Table 1).

**Table 1:** Median MAP and cardiac output (CO) measurements at each timepoint (*n* = 7).

	TP1	TP2	TP3	TP4
MAP (mmHg)	70 (68, 89)	44* (35, 80)	56 (52, 84)	69 (65, 84)
CO (L/min)	2.3 (1.4, 3.7)	0.6* (0.5, 0.9)	1.9 (1.0, 2.3)	2.2 (1.9, 4.8)

Normally distributed data are reported as mean ± standard deviation and range; Non-normally distributed data are presented as median (range) as denoted. ^*^Indicates significant decrease between TP1 and TP2. TP1, Anesthetized, post-instrumentation; TP2, Hemorrhagic shock; TP3, Re-transfusion (50% shed blood); TP4, Re-transfusion (100% shed blood); MAP, mean arterial blood pressure; CO, cardiac output.

CVC diameter at the left paralumbar view was significantly lower at TP2 (hemorrhagic shock) compared to TP1 after controlling for the effect of CO (*p* = 0.03) and the effect of MAP (*p* = 0.001) on the outcome variable. In other words, after controlling for the influence of CO and MAP, CVC was independently different at TP2 (hemorrhagic shock). Aortic diameter at the paralumbar view was significantly lower in both TP2 (hemorrhagic shock; p 0.002) and TP3 (50% re-transfusion; *p* = 0.023) compared to TP1 after controlling for CO. Similarly, aortic diameter was also significantly lower at TP2 (hemorrhagic shock) compared to TP1 (*p* = 0.001) and at TP3 (50% re-transfusion) compared to TP1 (*p* = 0.017) after adjusting for MAP (Table 2).

**Table 2:** Ao, CVCd, and CVC:Ao measurements at each timepoint (*n* = 7).

	TP1	TP2	TP3	TP4
CVC:Ao	0.94 ± 0.16	0.86 ± 0.18	0.96 ± 0.12	1.06 ± 0.07
CVCd (mm)	6.75 ± 1.28	4.63 ± 0.81	6.13 ± 1.16	7.41 ± 0.68
Ao (mm)	6.10 (6.7, 8.3)^†^	5.30 (4.0, 7.2)^†^	6.30 (5.6, 7.4)^†^	7.01 (6.4, 7.9)^†^

Normally distributed data are reported as mean ± standard deviation and range; Non-normally distributed data are presented as median (range) as denoted. ^†^Normality rejected. TP1, Anesthetized, post-instrumentation; TP2, Hemorrhagic shock; TP3, Re-transfusion (50% shed blood); TP4, Re-transfusion (100% shed blood); CVC:Ao, caudal vena cava to abdominal aorta ratio; CVCd, caudal vena cava diameter; Ao, abdominal aorta diameter.

**Figure 2:**
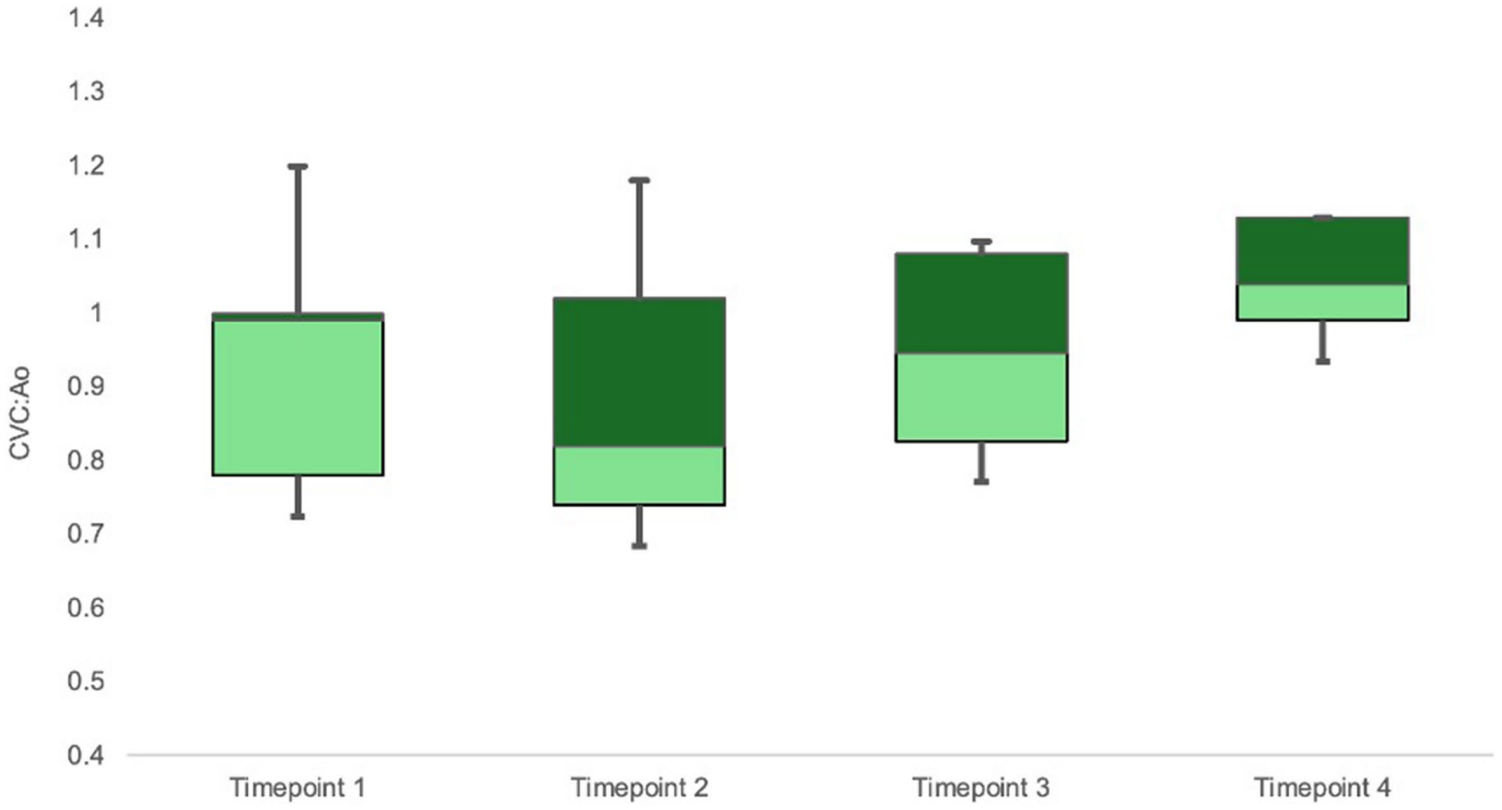
Box-whisker plot of CVC:Ao data for timepoints TP1-TP4 (baseline, hemorrhagic shock, 50% re-transfusion, and 100% re-transfusion). Center box lines indicate medians. Dark green box areas indicate 50th to 75th percentile values while light green box areas indicate 25th to 50th percentile values. Bars indicate minimum and maximum values.

CVC:Ao at TP1, TP2, TP3, and TP4 was 0.94 ± 0.16, 0.86 ± 0.18, 0.96 ± 0.12, and 1.06 ± 0.07, respectively (Figure 2). CVC:Ao was not significantly different between timepoints after correcting for CO and MAP (TP1 vs. TP2 *p* = 0.95, TP1 vs. TP3 *p* = 0.44, TP1 vs. TP4 *p* = 0.11 and TP1 vs. TP2 *p* = 0.19, TP1 vs. TP3 *p* = 0.93, TP1 vs. TP4 *p* = 0.07, respectively). CVC:Ao was not associated with measured changes in CO (*p* = 0.28) or MAP (*p* = 0.50).

Finally, all dogs were fluid responders with an increase in CO of >10% from hemorrhagic shock (TP2) to transfusion of 50% of shed blood (TP3) and from hemorrhagic shock (TP2) to full resuscitation (TP4). In contrast, only 5/7 dogs were fluid responders from 50 to 100% re-transfusion (TP3 to TP4).

## Discussion

In the current study, CVC diameter at the left paralumbar view is significantly smaller after induction of hemorrhagic shock consistent with a previous study investigating CVC diameter at a paralumbar view after 20 mL/kg blood donation (21). Studies in people show the CVC diameter is consistently smaller in hypovolemic people compared to euvolemic people and the maximal diameter of the cava during the respiratory cycle correlates well with catheter-based measures of right atrial pressure (22–24). Additionally, the aortic diameter was significantly different between baseline and hemorrhagic shock. Because both vessel diameters significantly decreased during severe hypovolemia, the CVC:Ao ratio did not significantly change with hypovolemia. Further studies are needed to determine a cut off value for paralumbar measurement of CVC diameter in dogs normalized to body weight that would accurately predict hypovolemia.

In our study, CVC diameter was not associated with changes in CO or MAP. Though previous studies have established that CVC measurements can predict fluid responsiveness (7, 25), this does not necessarily indicate that the degree of CVC diameter change corresponds to the degree of CO change. In states of volume loss, mean systemic filling pressure (MSFP) is bolstered by decreased capacitance of the vasculature and recruitment of unstressed volume, maintaining HR and CO (26). In this setting, compensation blunts the impact on CO. Additionally, CO is influenced by the complex interaction of many factors, including heart rate, stroke volume, preload, afterload, contractility, and autonomic nervous activity, among others (27) Similarly, MAP is affected by multiple factors, including systemic vascular resistance (SVR), CO, arterial compliance, and contractility, among others (1). It has been previously demonstrated that despite exsanguination, robust compensatory mechanisms will prevent a significant decrease in MAP (28) and due to tight regulation, MAP may not change despite an increase or decrease in CO (29). Therefore, the relationship between large vessel diameter, MAP, and CO is indirect and multifactorial, and may explain why changes in CVC and Ao measurements were not directly associated with changes in CO and MAP. The current study evaluated MAP and CO because MAP is a commonly employed, non-invasive static parameter, and direct CO measurement has not previously been utilized in a model of marked, known volume loss.

Prior veterinary studies documented a decreased CVC:Ao ratio in experimentally induced mild hypovolemia, such as volume loss secondary to furosemide administration with a mean 5.44% body weight reduction and 10 mL/kg blood donation (16, 19). In healthy dogs after a 10 mL/kg blood donation, the mean CVC:Ao ratio decreased significantly from 1.17 to 1.01 (19). In these studies, there was a significant decrease in CVC diameter with mild volume depletion, but not a concurrent significant change in aortic diameter (16, 21). In contrast, the current study induced a profound state of hypovolemia with approximately 40 mL/kg of blood loss, resulting in significant decreases of both vessel diameters and, as a result, no significant change in CVC:Ao ratio. Therefore, the CVC:Ao may significantly change in more mild states of hypovolemia, where the aortic diameter is not affected. The mean baseline CVC:Ao ratio in the current study was 0.94, which is comparable to a recent study in healthy dogs that established reference intervals for the CVC:Ao (0.93–1.32) (30). After inducing a state of profound hypovolemia in our study, the ratio was not significantly different at 0.86.

Further, discrepant CVC:Ao findings could be related to the type of shock employed and the duration. For example, a decreased CVC:Ao has been observed with simulated hypovolemic shock over hours (16), which may result in a different pathophysiologic response compared to the acute hemorrhagic shock model employed in the current study. With acute hemorrhagic shock, increased sympathetic tone and vasoconstriction are mainstays of compensation, while slow hypovolemic shock may have less drastic changes in vessel tone, relying on renal conservation of fluid via ADH and aldosterone (31). In addition to these differences, the slow hypovolemic model measured the CVC:Ao in awake patients, while our study performed measurements under general anesthesia. Though there is not literature evaluating the effect of anesthesia on CVC and Ao diameter, the known impact of anesthesia on vessel tone could affect the diameter of large vessels, potentially playing a role in the lack of CVC:Ao change observed in our study.

Two additional studies have evaluated the CVC:Ao in anesthetized animals at the porta hepatis from an intercostal view, evaluating the vessels at a location cranial to the paralumbar view. The first documented an increased CVC:Ao and good correlation with systolic pressure variation (SPV) in euvolemic dogs receiving an intravascular crystalloid fluid bolus (25). The second identified a decreased CVC:Ao, aortic diameter, and CVC diameter with hemorrhagic shock maintained for 30 min, followed by an increase in these measurements with volume resuscitation (20). Like the current study, CVC and Ao diameter were significantly affected by changes in volume, even under anesthesia in both studies. Discrepancies in CVC:Ao findings may suggest that in states of severe hypovolemia, the way in which CVC and aortic diameters change relative to each other is less predictable and perhaps influenced by differences in compensatory aortic tone.

Our study has several limitations. First, only 8 dogs were included in the study and images for one dog were lost and therefore not included in POCUS measurement analysis. The small sample size increases the likelihood of a type II error. This small sample size might explain why our CVC:Ao results are not consistent with other findings in the literature. Additionally, ultrasound measurements of the CVC and aorta have the inherent potential for error, as the accuracy of measuring a vessel is dependent on visualizing the vessel exactly on its midline and measuring diameter perpendicular to the vessel wall. Further, there is evidence in people that measurements of the vena cava are different in left lateral versus right lateral recumbency, with the smallest diameter measured in left lateral recumbency (32, 33). Therefore, it is possible that performing our measurements at a left paralumbar site, skewed the ratio of CVC diameter to aortic diameter. Our study also did not assess interobserver agreement, as all measurements and imaging were performed by the same individual. Interobserver agreement at this location should be further evaluated. The current study included young beagle dogs only, and therefore cannot evaluate the potential differences which might occur in various other breeds or ages.

The current hemorrhagic shock model produced profound hypovolemia through controlled blood loss and was able to assess the effect of hypovolemia on POCUS CVC diameter and CVC:Ao measurements. CVC diameter significantly decreased in states of hypovolemic shock independently of changes in CO. Further, changes in CVC diameter, aortic diameter, and CVC:Ao were not associated with changes in CO, emphasizing that CO is an indirect measurement of volume status and is more significantly affected by compensation.

## Data Availability

The raw data supporting the conclusions of this article will be made available by the authors, without undue reservation.

## Footnotes

^1^HemoSphere Advanced Monitoring Platform, Edwards Lifesciences Corp, Irvine, California.

^2^Sonoscape S9, SonoScape Medical Corp, Shenzhen, Guangdong, China.

^3^IntelliSpace Radiology Enterprise, Picture Archiving and Communication System (PACS), Philips Healthcare, Cambridge, MA.

^4^SAS v9.4, SAS Institute Inc., Cary, NC.

## References

[1] Boysen SR Gommeren K. Assessment of volume status and fluid responsiveness in small animals. Front Vet Sci. (2021) 8:630–43. doi: 10.3389/fvets.2021.630643 PMC8193042 34124213

[2] Beebe D. Assessing intravascular volume status and fluid responsiveness: a non-ultrasound approach In: Liebowitz AB, editor. Modern monitoring in anesthesiology and perioperative care. Cambridge: Cambridge University Press (2020). 109–16.

[3] Ochagavía A Baigorri F Mesquida J Ayuela JM Ferrándiz A García X. Hemodynamic monitoring in the critically patient. Recommendations of the Cardiological intensive care and CPR working Group of the Spanish Society of intensive care and coronary units. Med Intensiva. (2014) 38:154–69. doi: 10.1016/j.medin.2013.10.006, PMID: 24296336

[4] Monnet X Marik PE Teboul JL. Prediction of fluid responsiveness: an update. Ann Intensive Care. (2016) 6:111. doi: 10.1186/s13613-016-0216-7, PMID: 27858374 PMC5114218

[5] Darnis E Boysen S Merveille A Desquilbet L Chalhoub S Gommeren K. Establishment of reference values of the caudal vena cava by fast-ultrasonography through different views in healthy dogs. J Vet Intern Med. (2018) 32:1308–18. doi: 10.1111/jvim.15136, PMID: 29749656 PMC6060313

[6] Donati PA Guevara JM Ardiles V Guillemi EC Londoño L Dubin A. Caudal vena cava collapsibility index as a tool to predict fluid responsiveness in dogs. J Vet Emerg Crit Care. (2020) 30:677–86. doi: 10.1111/vec.13009, PMID: 33063922

[7] Rabozzi R Oricco S Meneghini C Bucci M Franci P. Evaluation of the caudal vena cava diameter to abdominal aortic diameter ratio and the caudal vena cava respiratory collapsibility for predicting fluid responsiveness in a heterogeneous population of hospitalized conscious dogs. J Vet Med Sci. (2020) 82:337–44. doi: 10.1292/jvms.19-0028, PMID: 31932519 PMC7118484

[8] Weekes AJ Tassone HM Babcock A Quirke DP Norton HJ Jayarama K. Comparison of serial qualitative and quantitative assessments of Caval index and left ventricular systolic function during early fluid resuscitation of hypotensive emergency department patients. Acad Emerg Med. (2011) 18:912–21. doi: 10.1111/j.1553-2712.2011.01157.x, PMID: 21906201

[9] Feissel M Michard F Faller JP Teboul JL. The respiratory variation in inferior vena cava diameter as a guide to fluid therapy. Intensive Care Med. (2004) 30:2233. doi: 10.1007/s00134-004-2233-5 15045170

[10] Menon L Balakrishnan J Wilson W Thomas M. Caval aortic index: a novel tool for fluid assessment in obstetric emergencies. J Emerg Trauma Shock. (2020) 13:50–3. doi: 10.4103/JETS.JETS_136_18, PMID: 32395050 PMC7204966

[11] Chen L Hsiao A Langhan M Riera A Santucci KA. Use of bedside ultrasound to assess degree of dehydration in children with gastroenteritis. Acad Emerg Med. (2010) 17:1042–7. doi: 10.1111/j.1553-2712.2010.00873.x 21040104 PMC3058669

[12] Barbier C Loubières Y Schmit C Hayon J Ricôme JL Jardin F. Respiratory changes in inferior vena cava diameter are helpful in predicting fluid responsiveness in ventilated septic patients. Intensive Care Med. (2004) 30:1740–6. doi: 10.1007/s00134-004-2259-8, PMID: 15034650

[13] AbdulGalil AE Abdelhalem AF Eldeeb AA. Role of inferior vena cava assessment in volume management in acute kidney injury patients. Egypt J Intern Med. (2024) 36:43. doi: 10.1186/s43162-024-00310-y

[14] Vieillard-Baron A Boissier F Slama M. Using echocardiography to predict fluid-responsiveness and manage the need for fluids. Intensive Care Med. (2024) 50:1137–42. doi: 10.1007/s00134-024-07407-6 38748267

[15] Caplan M Durand A Bortolotti P Colling D Goutay J Duburcq T. Measurement site of inferior vena cava diameter affects the accuracy with which fluid responsiveness can be predicted in spontaneously breathing patients: a post hoc analysis of two prospective cohorts. Ann Intensive Care. (2020) 10:168. doi: 10.1186/s13613-020-00786-1 33306164 PMC7732956

[16] Kwak J Yoon H Kim J Kim M Eom K. Ultrasonographic measurement of caudal vena cava to aorta ratios for determination of volume depletion in normal beagle dogs. Vet Radiol Ultrasound. (2018) 59:203–11. doi: 10.1111/vru.12566, PMID: 29024163

[17] Combet-Curt J Pouzot-Nevoret C Cambournac M Magnin M Nectoux A Bonnet-Garin JM. Ultrasonographic measurement of caudal vena cava to aorta ratio during fluid resuscitation of dogs with spontaneous circulatory shock. J Small Anim Pract. (2023) 64:669–79. doi: 10.1111/jsap.13654, PMID: 37452675

[18] Herreria-Bustillo VJ Fitzgerald E Humm KR. Caval-aortic ratio and caudal vena cava diameter in dogs before and after blood donation. J Vet Emerg Crit Care. (2019) 29:643–6. doi: 10.1111/vec.12900, PMID: 31625668

[19] Cambournac M Goy-Thollot I Violé A Boisvineau C Pouzot-Nevoret C Barthélemy A. Sonographic assessment of volaemia: development and validation of a new method in dogs: sonographic assessment of volaemia in dogs. J Small Anim Pract. (2018) 59:174–82. doi: 10.1111/jsap.12759, PMID: 28960319

[20] Azargoun R Avizeh R Ghadiri A Imani Rastabi H Pourmahdi M. Ultrasonographic assessment of caudal vena cava to aorta ratio as a novel endpoint in hemorrhagic shock resuscitation in dogs. Iran J Vet Surg. (2019) 14:125–32. doi: 10.22034/ivsa.2019.176714.1181

[21] Marshall KA Thomovsky EJ Brooks AC Johnson PA Lim CK Heng HG. Ultrasound measurements of the caudal vena cava before and after blood donation in 9 greyhound dogs. Can Vet J. (2018) 59:973–80. PMID: 30197440 PMC6091121

[22] Lee SL Daimon M Kawata T Kohro T Kimura K Nakao T. Estimation of right atrial pressure on inferior vena cava ultrasound in Asian patients. Circ J. (2014) 78:962–6. doi: 10.1253/circj.CJ-13-1234, PMID: 24476843

[23] Zhang J Zhao L. Volume assessment by inferior vena cava examination: bedside ultrasound techniques and practical difficulties. Curr Anesthesiol Rep. (2017) 7:416–20. doi: 10.1007/s40140-017-0232-7

[24] Dipti A Soucy Z Surana A Chandra S. Role of inferior vena cava diameter in assessment of volume status: a meta-analysis. Am J Emerg Med. (2012) 30:1414–1419.e1. doi: 10.1016/j.ajem.2011.10.017 22221934

[25] Meneghini C Rabozzi R Franci P. Correlation of the ratio of caudal vena cava diameter and aorta diameter with systolic pressure variation in anesthetized dogs. Am J Vet Res. (2016) 77:137–43. doi: 10.2460/ajvr.77.2.137, PMID: 27027706

[26] Magder S. Volume and its relationship to cardiac output and venous return. Crit Care. (2016) 20:271. doi: 10.1186/s13054-016-1438-7, PMID: 27613307 PMC5018186

[27] Cecconi M De Backer D Antonelli M Beale R Bakker J Hofer C. Consensus on circulatory shock and hemodynamic monitoring. Task force of the European Society of Intensive Care Medicine. Intensive Care Med. (2014) 40:1795–815. doi: 10.1007/s00134-014-3525-z, PMID: 25392034 PMC4239778

[28] Perel A Pizov R Cotev S. Systolic pressure variation is a sensitive indicator of hypovolemia in ventilated dogs subjected to graded hemorrhage. Anesthesiology. (1987) 67:498–502. doi: 10.1097/00000542-198710000-00009, PMID: 3310740

[29] Monge Garcia MI Guijo Gonzalez P Gracia Romero M Gil Cano A Oscier C Rhodes A. Effects of fluid administration on arterial load in septic shock patients. Intensive Care Med. (2015) 41:1247–55. doi: 10.1007/s00134-015-3898-7 26077088

[30] Barthélemy A Combet-Curt J Dupanloup A Gillet B Cambournac M Bonnet-Garin JM. Establishment of reference intervals for caudal vena cava-to-aorta ratio measured Ultrasonographically in healthy nonsedated dogs. Top Companion Anim Med. (2023) 56-57:100822. doi: 10.1016/j.tcam.2023.100822 37802246

[31] Valverde A. Fluid resuscitation for refractory hypotension. Front Vet Sci. (2021) 8:621696. doi: 10.3389/fvets.2021.621696, PMID: 33778035 PMC7987676

[32] Nakao S Come PC McKay RG Ransil BJ. Effects of positional changes on inferior vena caval size and dynamics and correlations with right-sided cardiac pressure. Am J Cardiol. (1987) 59:125–32. doi: 10.1016/S0002-9149(87)80084-X, PMID: 3812222

[33] Ciozda W Kedan I Kehl DW Zimmer R Khandwalla R Kimchi A. The efficacy of sonographic measurement of inferior vena cava diameter as an estimate of central venous pressure. Cardiovasc Ultrasound. (2015) 14:33. doi: 10.1186/s12947-016-0076-1, PMID: 27542597 PMC4992235

